# Antitumor Effects of Orally Administered Rare Sugar D-Allose in Bladder Cancer

**DOI:** 10.3390/ijms23126771

**Published:** 2022-06-17

**Authors:** Yoichiro Tohi, Rikiya Taoka, Xia Zhang, Yuki Matsuoka, Akihide Yoshihara, Emi Ibuki, Reiji Haba, Kazuya Akimitsu, Ken Izumori, Yoshiyuki Kakehi, Mikio Sugimoto

**Affiliations:** 1 Department of Urology, Faculty of Medicine, Kagawa University, 1750-1 Ikenobe, Miki-cho 761-0793, Japan; tohi.yoichiro@kagawa-u.ac.jp (Y.T.); cho.ka@kagawa-u.ac.jp (X.Z.); matsuoka.yuki@kagawa-u.ac.jp (Y.M.); president-yk@kagawa-u.ac.jp (Y.K.); sugimoto.mikio@kagawa-u.ac.jp (M.S.); 2 International Institute of Rare Sugar Research and Education, Kagawa University, 2393 Ikenobe, Miki-cho 761-0795, Japan; yoshihara.akihide@kagawa-u.ac.jp (A.Y.); akimitsu.kazuya@kagawa-u.ac.jp (K.A.); izumori@izumoring.com (K.I.); 3 Department of Diagnostic Pathology, Faculty of Medicine, Kagawa University, 1750-1 Ikenobe, Miki-cho 761-0793, Japan; ibuki.emi@kagawa-u.ac.jp (E.I.); haba.reiji@kagawa-u.ac.jp (R.H.)

**Keywords:** bladder cancer, D-allose, rare sugar, reactive oxygen species, thioredoxin-interacting protein

## Abstract

D-allose is a rare sugar that has been reported to up-regulate thioredoxin-interacting protein (TXNIP) expression and affect the production of intracellular reactive oxygen species (ROS). However, the antitumor effect of D-allose is unknown. This study aimed to determine whether orally administered D-allose could be a candidate drug against bladder cancer (BC). To this end, BC cell lines were treated with varying concentrations of D-allose (10, 25, and 50 mM). Cell viability and intracellular ROS levels were assessed using cell viability assay and flow cytometry. TXNIP expression was evaluated using Western blotting. The antitumor effect of orally administered D-allose was assessed using a xenograft mouse model. D-allose reduced cell viability and induced intracellular ROS production in BC cells. Moreover, D-allose stimulated TXNIP expression in a dose-dependent manner. Co-treatment of D-allose and the antioxidant L-glutathione canceled the D-allose-induced reduction in cell viability and intracellular ROS elevation. Furthermore, oral administration of D-allose inhibited tumor growth without adverse effects (*p* < 0.05). Histopathological findings in tumor tissues showed that D-allose decreased the nuclear fission rate from 4.1 to 1.1% (*p* = 0.004). Oral administration of D-allose suppressed BC growth in a preclinical mouse model, possibly through up-regulation of TXNIP expression followed by an increase in intracellular ROS. Therefore, D-allose is a potential therapeutic compound for the treatment of BC.

## 1. Introduction

Bladder cancer (BC) is the most common genitourinary cancer and the 10th most common cancer worldwide, with an estimated 573,278 new cases reported in 2020 [1]. However, unlike other cancers, there has been no improvement in the survival rates of patients with BC since the 1990s [2]. In clinical practice, nearly one-third of patients diagnosed with BC cannot be cured, and over 200,000 patients die from this disease each year worldwide [1]. Urothelial carcinoma is the most common type of BC. Despite recent advances in treatment options, locally advanced and metastatic urothelial carcinoma (mUC) has a poor prognosis [3]. Therefore, a novel class of drugs for patients with mUC is urgently required.

According to the International Society of Rare Sugars, rare sugars are monosaccharides found only in small amounts in nature and there are more than 50 types of rare sugars [4]. D-allose, the C3 epimer of D-glucose, is a rare sugar that has been approved as a food product in Japan. To date, no adverse events related to D-allose consumption have been reported. Recently, D-allose has attracted attention due to its various physiological functions, which differ from those of D-glucose. One of these functions is the suppression of cancer growth in various types of cancer [5,6,7]. However, the antitumor effects of orally administered D-allose are unknown, and no antitumor activity against BC has been reported so far.

It has recently been reported that D-allose induces the expression of thioredoxin-interacting protein (TXNIP) in several types of cancer [5,6,7]. TXNIP, also known as thioredoxin-binding protein-2 or vitamin D3 up-regulated protein-1, interacts with thioredoxin [8]. Thioredoxin, a protein disulfide oxidoreductase, is a potent antioxidant [9]. TXNIP has been reported to induce the production of intracellular reactive oxygen species (ROS) and arrest the cell cycle by decreasing the amount of active thioredoxin; hence, it has attracted attention as a potential therapeutic target [10]. Thus, we aimed to investigate the antitumor effect of the rare sugar, D-allose, on BC cells and its potential clinical efficacy as an oral treatment for mUC patients, using a preclinical mouse model.

## 2. Results

### 2.1. D-Allose Inhibits Bladder Cancer Cell Viability

**Figure 1:**
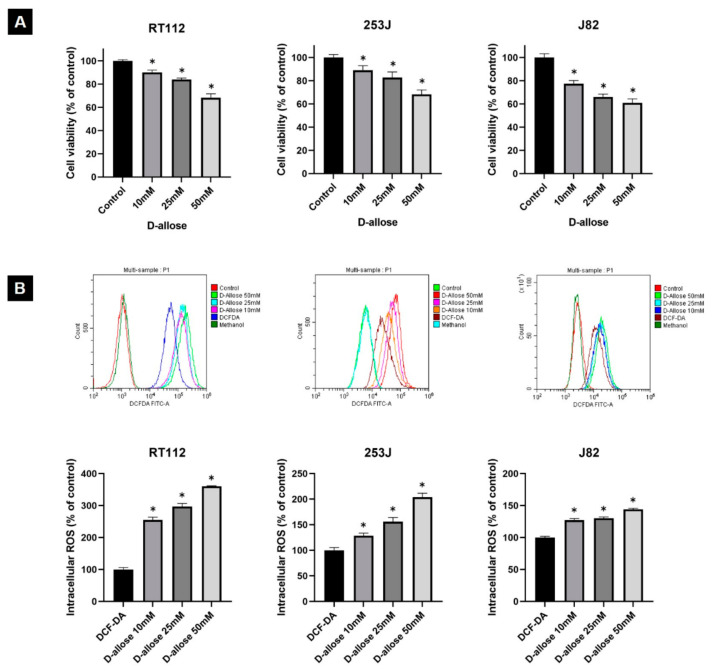
D-allose inhibits cell viability and induces intracellular reactive oxygen species (ROS) in three bladder cancer cell lines. (**A**) An MTT assay reveals that treatment with varying concentrations of D-allose for 24 h inhibits the cell viability of RT112, 253J, and J82 bladder cancer cell lines. Statistically significant (*p* < 0.05) decreases in cell viability rates compared with the control (set at 100%) are represented by asterisks (*). (**B**) The 2′,7′-dichlorofluorescindiacetate (DCF-DA) fluorescence-activated cell sorting (FACS) analysis revealed that treatment with varying concentrations of D-allose for 1 h induced intracellular ROS production in RT112, 253J, and J82 bladder cancer cell lines. Statistically significant (*p* < 0.05) increases in intracellular ROS levels compared with the control (DCF-DA; set at 100%) are represented by asterisks (*).

The MTT assay revealed that D-allose inhibited the viability of all three BC cell lines (RT112, 253J, and J82) in a dose-dependent manner. Treatment with 50 mM D-allose for 24 h significantly decreased the viability rates of RT112, 253J, and J82 cells to 68.4 ± 1.9, 68.2 ± 2.2, and 60.9 ± 3.4%, respectively, compared to the control cells (*p* < 0.0001, *p* = 0.0003, and *p* = 0.0012, respectively) (Figure 1A).

### 2.2. D-Allose Induces Intracellular ROS and Stimulates the TXNIP Expression in Bladder Cancer Cells

**Figure 2:**
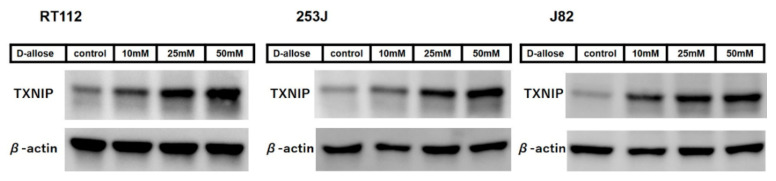
D-allose up-regulated thioredoxin binding protein (TXNIP) in three bladder cancer cell lines at 48 h post-treatment with varying concentrations of D-allose (10, 25, and 50 mM). Western blotting revealed that D-allose up-regulates TXNIP expression in RT112, 253J, and J82 bladder cancer cell lines in a dose-dependent manner. β-actin served as the loading control.

The fluorescence-activated cell sorting (FACS) analysis showed a significant dose-dependent increase in intracellular ROS levels following D-allose treatment in all three BC cell lines (Figure 1B). After 1 h of 50 mM D-allose treatment, the intracellular ROS levels were increased by 360.2 ± 1.7, 203.8 ± 7.9, and 144 ± 1.8% in RT112, 253J, and J82, respectively, compared with the controls (*p* < 0.0001, *p* = 0.0004, and *p* < 0.0001, respectively). Western blotting revealed that D-allose stimulated TXNIP expression in all three BC cell lines in a dose-dependent manner (Figure 2).

### 2.3. Suppression of D-Allose-Induced Antitumor Effects by Antioxidant Glutathione

**Figure 3:**
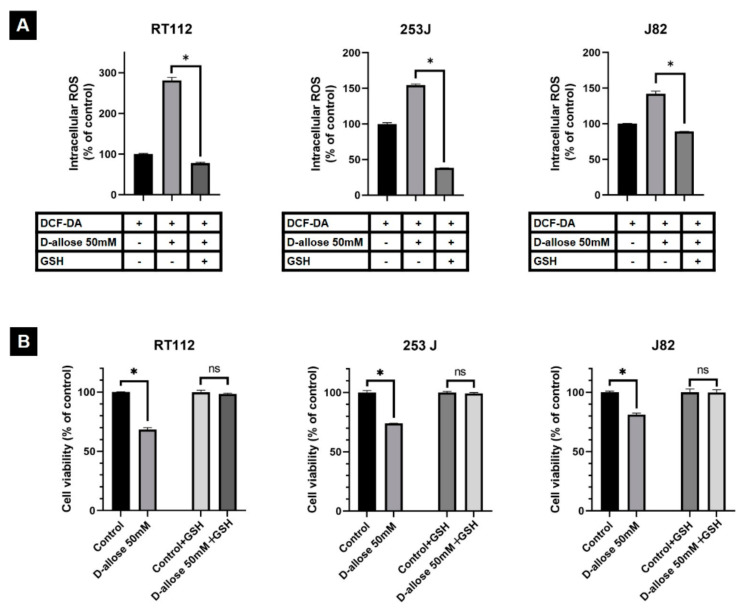
Co-treatment of D-allose and antioxidant glutathione (GSH) suppressed the D-allose-induced intracellular reactive oxygen species (ROS) elevation and cell viability inhibition in three bladder cancer cell lines. (**A**) A fluorescence-activated cell sorting (FACS) analysis showed a significant decrease in intracellular ROS at 1 h post-treatment of 50 mM D-allose with GSH in RT112, 253J, and J82, respectively, compared with the control (2′,7′-dichlorofluorescindiacetate [DCF-DA]; **A**). Statistically significant (*p* < 0.05) increases in intracellular ROS rates compared with the control (set at 100%) are represented by asterisks (*). (**B**) The MTT assay revealed that the antioxidant GSH reduced the inhibition of RT112, 253J, and J82 bladder cancer cell viability by D-allose. Statistically significant (*p* < 0.05) decreases in cell viability rates compared with the control (set at 100%) are represented by asterisks (*). ns: not significant.

The FACS analysis showed that D-allose-induced intracellular ROS production was significantly attenuated by co-treatment with the antioxidant glutathione (GSH) to −203.2 ± 4.8 (*p* < 0.0001), −116.1 ± 1.1 (*p* < 0.0001), and −53 ± 2.2% (*p* < 0.0001) in RT112, 253J, and J82 cells, respectively (Figure 3A). As a result, intracellular ROS levels in cells that were co-treated with D-allose and GSH were similar to or lower than those in the control cells. In addition, the antioxidant GSH suppressed D-allose-induced cancer cell viability inhibition. The MTT assay revealed that cell viability under co-treatment with D-allose and GSH was 98.5 ± 0.6 (*p* = 0.45), 99.4 ± 0.4 (*p* = 0.43), and 99.9% ± 1.3% (*p* = 0.98) for RT112, 253J, and J82, respectively, compared to that of the controls (Figure 3B).

### 2.4. Tumor Growth Inhibition in a Preclinical Mouse Model by Oral Administration of D-Allose

**Figure 4:**
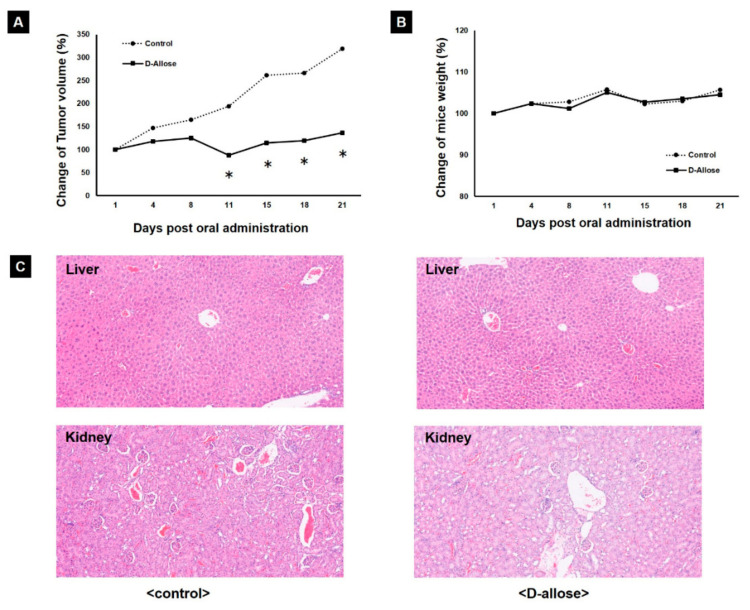
In vivo experiment. (**A**) Oral administration of D-allose inhibited tumor growth in vivo in mouse xenografts. RT112 tumors, treated with oral administration of D-allose or normal saline, were enucleated 20 days after treatment initiation. Statistically significant (*p* < 0.05) decreases in tumor volume compared with the control (set at 100%) are represented by asterisks (*). (**B**) Mouse xenografts treated with oral D-allose showed no significant difference in body weight from that in the control (normal saline). (**C**) There was no difference in the histological findings of the kidneys or livers between mice treated with oral administration of D-allose and those treated with normal saline.

Orally administered D-allose inhibited tumor growth in a preclinical mouse model. There was a significant difference in tumor volumes between D-allose-treated mice and normal saline-treated mice after a 15-day treatment period (Figure 4A; *p* < 0.05). Histological findings in tumor tissues showed that D-allose decreased the number of cancer cells with nuclear fission from 4.1 to 1.1% (*p* = 0.004). In addition, D-allose did not affect body weight (Figure 4B). Furthermore, there were no differences in the histological findings of the kidney and liver tissues between mice treated with oral D-allose and those treated with normal saline (Figure 4C).

## 3. Discussion

The rare sugar D-allose may be a novel drug candidate for patients with mUC. This study revealed the antitumor activity of D-allose in BC cells, which is in part due to the up-regulation of TXNIP and the subsequent increase in intracellular ROS levels. In addition, we demonstrated that orally administered D-allose suppressed BC growth without obvious adverse effects in a preclinical xenograft mouse model.

TXNIP expression is down-regulated in several cancers [11,12,13]. Reportedly, in BC, TXNIP is down-regulated in cancer cells according to grade and stage, and loss of TXNIP expression promotes BC progression [14]. TXNIP plays a vital role in redox homeostasis; it binds to the active cysteine residue of thioredoxin and inhibits its antioxidant function [15,16], resulting in increased intracellular ROS production [8,17]. Several agents have been reported to kill BC cells by inducing excessive intracellular ROS production [18,19]. In the present in vitro study, Western blotting analysis indicated that D-allose stimulated TXNIP expression, consistent with previous studies on other cancer types [5,6,7], and the FACS analysis revealed the up-regulation of intracellular ROS levels after treatment with D-allose. In addition, co-treatment of D-allose and the antioxidant GSH suppressed D-allose-induced intracellular ROS elevation and cell viability inhibition in BC cells. These results suggest that the TXNIP production induced by D-allose is a key factor in its antitumor effects through intracellular ROS production.

A key finding of this study was that the oral administration of D-allose inhibited BC growth in a xenograft mouse model. Although previous in vivo studies on D-allose have demonstrated its antitumor effect via injection around the tumor [5,7], this study is the first to reveal the antitumor effect of the oral administration of D-allose. Histologically, orally administered D-allose significantly decreases the nuclear fission of BC cells. TXNIP reduces cancer cell viability by arresting the cell cycle [10]. Therefore, the suppression of nuclear fission may contribute to the reduction in BC cell viability by D-allose through TXNIP. As D-allose is available as a powder, oral administration is a clinically relevant method. Further studies need to be performed using orthotopic xenograft models with tumor microenvironments, similar to those of the original tumor.

In this study, D-allose had no apparent adverse effects on the kidneys, liver, or body weight in the mouse xenograft model, similar to the findings in a previous study of other cancer types using a D-allose injection around the tumor [20]. Our safety data from the oral administration of D-allose can promote its clinical application. In addition, a pharmacokinetic study of D-allose, conducted at the time of approval for Japanese food materials, showed that most D-allose was excreted in the urine in the short term. Therefore, urinary excretion of D-allose has the potential to inhibit the progression of mUC and prevent recurrence of non-muscle-invasive BC (NMIBC) in terms of clinical application. The main current standard treatment for the prevention of NMIBC recurrence is intravesical instillation of chemotherapeutic agents and bacillus Calmette–Guerin, but it is difficult to continue treatment for an adequate duration due to their adverse effects [21]. In this context, D-allose may be a solution for BC treatment.

This study had some limitations. Firstly, we investigated the antitumor effect of oral administration of D-allose using a xenograft mouse model. However, since the metabolism of D-allose and the growth environment of BC cells may differ between rodents and humans, it is possible that the antitumor effect of orally administered D-allose may not be observed in humans. However, the safety of D-allose consumption has already been confirmed. Further studies are needed to determine whether oral administration of D-allose has clinical utility for BC in humans. Secondly, this study did not elucidate the mechanism of D-allose-induced TXNIP up-regulation, followed by intracellular ROS elevation. After D-allose-induced TXNIP up-regulation in hepatocellular carcinoma was revealed by microarray analysis [6], it was reported in various cancers, including in this study [5,7]. In addition, it was reported that TXNIP binds to the active cysteine residue of thioredoxin and inhibits its antioxidative function [22]. In this context, intracellular ROS elevation via inhibition of thioredoxin by TXNIP is definitive according to a previous report [10]. However, the mechanism of TXNIP induction through a 1-h D-allose treatment is poorly understood and requires further investigation. Thirdly, in the in vivo xenograft experiment, D-allose was orally administered at 400 mg/kg. We set this D-allose concentration because it provided a favorable balance between the safety and effectiveness of the treatment, based on a previous report [23]. The results of intraperitoneal administration of D-allose were used as evidence due to the lack of data on the oral administration of D-allose. However, in our study, we believe that the dose of 400 mg/kg D-allose was within the appropriate dosage range, as it has led to successful antitumor effects in the xenograft mouse model.

## 4. Materials and Methods

### 4.1. Reagents and Cell Lines

D-allose was supplied by the International Institute of Rare Sugar Research and Education of Kagawa University (Kagawa, Japan). β-actin (ab8227; Abcam, Cambridge, UK) and anti-TXNIP (#14715; Cell Signaling Technology, Inc., Beverly, MA, USA) were used as the primary antibodies, and horseradish peroxidase-conjugated anti-rabbit IgG (ab6721; Abcam, Cambridge, UK) was used as the secondary antibody. DCF-DA (D6883) and reduced GSH (G6013) were obtained from Sigma-Aldrich (St. Louis, MO, USA). In addition, human BC cell lines (RT112: low grade urothelial carcinoma; 253J and J82: high grade urothelial carcinoma) were obtained from the Japan Cancer Research Bank (Tokyo, Japan) and cultured in RPMI-1640 (Wako, Osaka, Japan). They were supplemented with a 10% fetal bovine serum (FBS) (Sigma-Aldrich), a HEPES solution (Sigma-Aldrich), and penicillin-streptomycin (Thermo Fisher Scientific, Waltham, MA, USA). At the time of the study on ROS, E-MEM (Wako, Osaka, Japan) with 10% FBS and penicillin-streptomycin were used. All cancer cells were incubated at 37 °C in a 5% CO_2_ atmosphere.

### 4.2. Cell Viability Assay

The in vitro cell viability inhibition test was performed using the 3-(4,5-demerthylthiazol-2-yl)-2,5-diphenyltetrazolium bromide (MTT) assay. Briefly, RT112, 253J, and J82 cells were seeded in 96-well plates at a concentration of 3–5 × 10^3^ cells/well, depending on the proliferation rate of the cell line, and incubated for 24 h. The cells were treated with different concentrations (10, 25, and 50 mM) of D-allose for 24 h. The control group contained the same amount of RPMI-1640 instead of D-allose. Cell viability was assessed by the MTT assay using a Cell Proliferation Kit I (Roche, Mannheim, Germany). Cell viability in each well was measured in terms of optical density at a wavelength of 570 nm, with 750 nm as the reference wavelength. All measurements were repeated at least in triplicate.

### 4.3. Intracellular ROS Evaluation

We performed a FACS analysis to evaluate the effect of D-allose on intracellular ROS levels in BC cell lines (RT112, 253J, and J82). Cells were plated in a 100-mm dish at 3–5 × 10^3^ cells/dish, depending on the proliferation rate of the cell line. After 24 h of incubation, cells were treated with methanol or varying concentrations of D-allose (10, 25, and 50 mM) for 1 h. D-allose-induced intracellular ROS production was estimated by adding DCF-DA (10 µM). Intracellular ROS levels were measured by FACS using a CytoFLEX S (Beckman Coulter, CA, USA). All measurements were repeated in triplicate, and the data were analyzed using CytExpert software (Beckman Coulter, Brea, CA, USA).

### 4.4. Co-Treatment with D-Allose and GSH

A FACS analysis was performed to evaluate the effect of D-allose, with and without GSH, on intracellular ROS levels in BC cells. After 24 h of incubation, the BC cells (RT112, 253J, and J82) were treated with 50 mM D-allose, with and without 5 mM GSH, for 1 h. Intracellular ROS levels were analyzed as described above.

In addition, we evaluated the effects of D-allose, with and without the antioxidant GSH, on cell viability using the MTT assay. After 24 h of incubation, the BC cells (RT112, 253J, and J82) were treated with 50 mM D-allose, with and without 5 mM GSH, for 24 h. Cell viability was analyzed as described above.

### 4.5. Western Blotting

Western blotting was performed to evaluate the effects of D-allose on the expression in BC cells. After 24 h of incubation, the cells (RT112, 253J, and J82) were treated with varying concentrations of D-allose (10, 25, and 50 mM) for 48 h. Both attached and floating cells were harvested and lysed. Samples were run on a 10% Mini-PROTEAN TGX Precast Gel (Bio-Rad Laboratories Inc. Hercules, CA, USA), transferred onto polyvinylidene difluoride membranes, and blocked in a SuperBlock Blocking Buffer (Thermo Fisher Scientific, Waltham, MA, USA) for 1 h. Anti-TXNIP (1:1000) or β-actin (1:1000) were used as the primary antibodies to react with the iBind Flex Western System (Thermo Fisher Scientific). Signals were developed using an ECL (enhanced chemiluminescence) kit (Amersham Biosciences, Buckinghamshire, UK).

### 4.6. In Vivo Xenograft Experiment

Xenografts were prepared by subcutaneously implanting tumors derived from RT112 cells into the backs of 5-week-old female nude mice (BALB/cA Jcl-nu/nu; CLEA, Japan). A total of 17 nude mice with tumor volumes reaching 100 mm^3^ were randomly divided into a control group of 8 mice and a D-allose treatment group of 9 mice. Mice in the control and D-allose treatment groups were orally administered a daily dose of 0.2 mL normal saline or 400 mg/kg D-allose dissolved in normal saline, using a disposable feeding needle (FG5202K; Kenis, Osaka, Japan). Changes in mouse body weight and tumor volume were monitored every 3 or 4 days using a digital scale and caliper, respectively. Tumor volume was calculated using the following formula: (length) × (width)^2^ × 0.5. After 20 days of treatment, the tumor, kidneys, and liver were excised individually.

All tissues were fixed in 4% phosphate-buffered paraformaldehyde, embedded in paraffin, and dissected into 4 μm-thick sections for hematoxylin-eosin staining. The stained sections were used to evaluate the fission rate of cancer cells using a Nikon Eclipse 55i microscope with a standard 22-mm-diameter eyepiece (Nikon, Konan, Tokyo, Japan). A pathologist evaluated all measurements using 3 sections, and the mean value was considered for analysis. Animal experiments were performed in accordance with the Guide for the Care and Use of Laboratory Animals of Kagawa University (No: 19637-1).

### 4.7. Statistical Analysis

A *p*-value of <0.05 was considered to indicate statistical significance. The Student’s t-test was used to compare data between the two groups. Data are presented as mean ± standard error (SE). All statistical analyses were performed using SPSS for Windows (version 25.0., IBM Corp., Armonk, NY, USA).

## 5. Conclusions

This study illustrated that oral administration of D-allose suppresses BC growth in a preclinical mouse model. It also revealed that the up-regulation of TXNIP expression, followed by an increase in intracellular ROS production, is a possible mechanism of the antitumor effect of D-allose. Thus, D-allose is a potential therapeutic compound for the treatment of BC, and further research on its efficacy and mode of action is warranted.

## Data Availability

Not applicable.
